# Evidence that pericytes regulate aquaporin-4 polarization in mouse cortical astrocytes

**DOI:** 10.1007/s00429-013-0629-0

**Published:** 2013-08-28

**Authors:** Georg Andreas Gundersen, Gry Fluge Vindedal, Øivind Skare, Erlend A. Nagelhus

**Affiliations:** 1 Centre for Molecular Medicine Norway, Nordic EMBL Partnership, University of Oslo, P.O. Box 1137, Blindern, 0318 Oslo, Norway; 2 Letten Centre and Department of Physiology, Institute of Basic Medical Sciences, University of Oslo, 0317 Oslo, Norway; 3 National Institute of Occupational Health, 0033 Oslo, Norway; 4 Department of Neurology, Oslo University Hospital, Rikshospitalet, 0027 Oslo, Norway; 5 Department of Neurosurgery, Center for Translational Neuromedicine, University of Rochester Medical Center, Rochester, NY 14642 USA

**Keywords:** AQP4, Endfeet, Glia, Blood–brain barrier, Syntrophin, Water channel

## Abstract

Aquaporin-4 (AQP4) water channels are concentrated in astrocytic endfoot membranes at the brain–blood and brain–cerebrospinal fluid interfaces. The mechanisms underpinning the polarized distribution of AQP4 are poorly understood. Here we tested the hypothesis that pericytes regulate AQP4 anchoring to perivascular astrocytic endfoot membranes. AQP4 immunofluorescence of brain sections obtained from novel transgenic double reporter mice expressing enhanced green fluorescent protein (eGFP) in astrocytes and Discoma Red (DsRed) in pericytes revealed strong AQP4 signal in astrocytic processes adjacent to pericytes. Quantitative immunogold analysis of C57BL/6 mice showed that the AQP4 expression was higher in endfoot membranes abutting pericytes than in those facing endothelial cells. Similar findings were made for α-syntrophin, a member of the dystrophin-associated protein complex (DAPC). The enrichment of α-syntrophin in membranes ensheathing pericytes persisted after *Aqp4* gene deletion. Our data support the concept that pericytes regulate AQP4 polarization.

## Introduction

AQP4 regulates water transport across the blood–brain barrier (BBB) and along paravascular pathways (Haj-Yasein et al. 2011; Iliff et al. 2012). Early immunogold studies revealed that AQP4 has a polarized distribution with tenfold higher density in glial endfoot membranes than in other membrane domains (Nagelhus et al. 1998; Nielsen et al. 1997). Later it was shown that the DAPC is essential for AQP4 polarization in glia. Notably, mice deficient in dystrophin (Frigeri et al. 2001; Enger et al. 2012), α-syntrophin (Neely et al. 2001; Eilert-Olsen et al. 2012), dystroglycan (Noell et al. 2011), or α-dystrobrevin (Lien et al. 2012) show reduced perivascular AQP4 expression. The DAPC is attached to the perivascular basal lamina through binding between the α-subunit of dystroglycan and the extracellular matrix components laminin and agrin (Satz et al. 2010). Hence, loss of agrin reduces AQP4 expression in glial endfeet surrounding blood vessels (Rauch et al. 2011).

The concept that vascular cells regulate AQP4 anchoring was bolstered by the discovery that pericyte-deficient mice showed abnormal AQP4 expression around blood vessels (Armulik et al. 2010). However, the mutant mice used in this study had abnormal vessel diameter, disrupted BBB, and increased brain water content, which could have indirect effects on the subcellular AQP4 distribution. In the present study, we used mice with intact BBB and investigated whether AQP4 anchoring in astrocytic endfeet depends on their intimate relationship to pericytes.

## Materials and methods

### Animals

Male wild-type (C57BL/6, Jackson Laboratories, Boulder, CO), *Aqp4* ^−*/*−^ (Thrane et al. 2011), *Snta1* ^−/−^ (lacking the gene for α-syntrophin) (Adams et al. 2000), and *GLT1*-eGFP/*NG2*-DsRed transgenic double reporter mice at 8–18 weeks of age were used in this study. The latter animals were generated by crossing bacterial artificial chromosome (BAC) promoter reporter transgenic mice that express the fluorescent proteins eGFP and DsRed under the control of the natural GLT1 (Regan et al. 2007) and NG2 BAC promoters (Zhu et al. 2008), respectively. The animals were allowed ad libitum access to food and drinking water. All experiments comply with Norwegian laws and were approved by the Animal Care and Use Committee of Institute of Basic Medical Sciences, University of Oslo.

### Fixation

The animals were deeply anesthetized with a mixture of chloral hydrate, magnesium sulfate and pentobarbital (142, 70 and 32 mg/kg body weight i.p., respectively) before transcardiac perfusion (flow rate ~8 ml/min) with 2 % dextran in 0.1 M phosphate buffer (PB; pH 7.4) for 15–20 s and either 4 % formaldehyde in PB for 15 min (for immunofluorescence experiments; 3 *GLT1*-eGFP/*NG2*-DsRed transgenic double reporter mice were used), 4 % formaldehyde and 0.1 % glutaraldehyde in PB for 20 min (for AQP4 immunogold cytochemistry; 5 wild-type and 5 *Aqp4* ^−/−^ mice were used), or bicarbonate-buffered 4 % formaldehyde containing 0.2 % picric acid at pH 6.0, followed by a similar fixative at pH 10.0 (“pH-shift” protocol) (Nagelhus et al. 1998) (for α-syntrophin immunogold cytochemistry; 5 wild-type, 5 *Aqp4* ^−/−^, and 3 *Snta1* ^−/−^ mice were used).

### Light microscopic immunocytochemistry

The perfused animals were stored at 4 °C overnight. The brain was removed and cryoprotected in sucrose (10, 20 and 30 % in PB), and sections were cut at 16 μm on a cryostat. Light microscopic immunocytochemistry was carried out using an indirect fluorescence method (Nagelhus et al. 1998). The primary anti-AQP4 antibody (Sigma, 2 μg/ml) was revealed by donkey secondary antibodies with indodicarbocyanine (Cy5, Jackson ImmunoResearch Laboratories, Inc., West Grove, PA; 1:1,000). Cortical sections were viewed and photographed with a Zeiss LSM 5 PASCAL microscope equipped with epifluorescence optics, using filter BP 505–530, LP 560 and LP 650, and objective 20×/0.75 Plan-Apochromat, 40×/1.3 Oil Plan-Neofluar, or 63×/1.40 Oil Plan-Apochromat.

### Embedding and electron microscopic cytochemistry

Small blocks of fixed cortex were subjected to freeze substitution as described previously (Nagelhus et al. 1998). In brief, the specimens were cryoprotected by immersion in graded concentrations of glycerol (10, 20, and 30 %) in PB and plunged into liquid propane (−170 °C) in a cryofixation unit (KF 80; Reichert, Wien, Austria). The samples were then immersed in 0.5 % uranyl acetate dissolved in anhydrous methanol (−90 °C) in a cryosubstitution unit (AFS; Reichert). The temperature was raised in steps of 4 °C/h to −45 °C. Samples were washed with anhydrous methanol and infiltrated with Lowicryl HM20 resin at −45 °C with a progressive increase in the ratio of resin to methanol. Polymerization was carried out with UV light (360 nm) for 48 h. Ultrathin sections were cut with a Reichert ultramicrotome, mounted on nickel grids, and processed for immunogold cytochemistry as described previously (Nagelhus et al. 1998). Briefly, sections were incubated sequentially in the following solutions (at room temperature): (1) 50 mM glycine in Tris buffer (5 mM) containing 0.01 % Triton X-100 and 50 mM NaCl (TBST; 10 min); (2) 0.2 % milk powder in TBST (10 min); (3) primary antibodies (AQP4, amino acid residues 249–323, Milipore, 1.5 μg/ml; α-syntrophin (Ab Syn259) (Peters et al. 1997), 1.2 μg/ml) diluted in the solution used in the preceding step (overnight); (4) same solution as in step 1 (10 min ×2); (5) same solution as in step 2 (10 min); (6) gold-conjugated IgG (GAR10 nm; Abcam), diluted 1:20 in TBST containing 2 % human serum albumin or 0.2 % milk powder and polyethylene glycol (0.5 mg/ml, 1 h). Finally, the sections were counterstained and examined in a Fei Tecnai 12 transmission electron microscope.

### Detection and quantification of gold particles

The analyzer was blind to genotypes. Digital images were acquired with a commercially available image analysis program (“analySIS” Soft Imaging Systems GmbH, Münster). The program had been modified for acquisition of high-resolution digital images and semiautomatic evaluation of immunogold-labeled cellular volumes and surfaces (membranes). For the present purpose, images of membrane segments were recorded at a nominal magnification of ×43.000, in 2.048 × 2.048 (8-bit) images. All membrane segments that could be identified as belonging to perivascular endfeet of astrocyte cells were imaged. Membrane segments of interest were drawn in the overlay and assigned a type label. Gold particles in the neighborhood of each membrane curve were detected semiautomatically, and the distance between each particle’s center of gravity and its membrane curve was calculated by the program. All images, with associated curves, particles, and measurements, were saved to allow later verification and correction. Further analyses were done in analySIS. Particles localized within 23.5 nm from their membrane curve were included in the automated calculation of the number of particles per unit length of membrane (linear particle density). The measurements were exported to the SPSS 18.0 for Mac software package (SPSS, Chicago, IL) for survey and quality control.

### Statistical analysis of immunogold data

We used Poisson mixed models for the analyses of the gold particle counts in the AQP4 and α-syntrophin data. These models take into account the dependency between observations by including nested variance terms. Three variance terms were included, one for between animal variation, one for between membrane domain variation, and one for within membrane domain variation. These models were analyzed by the R function glmer in the lme4 package, which gave us estimated differences in linear particle density between membrane domains (endfoot membranes facing endothelial cells and pericytes, respectively) with corresponding *P* values. In the AQP4 analysis, we allowed the within membrane variation to differ between membranes. The chosen model structures were based on likelihood ratio tests (Enger et al. 2012).

## Results

**Fig. 1:**
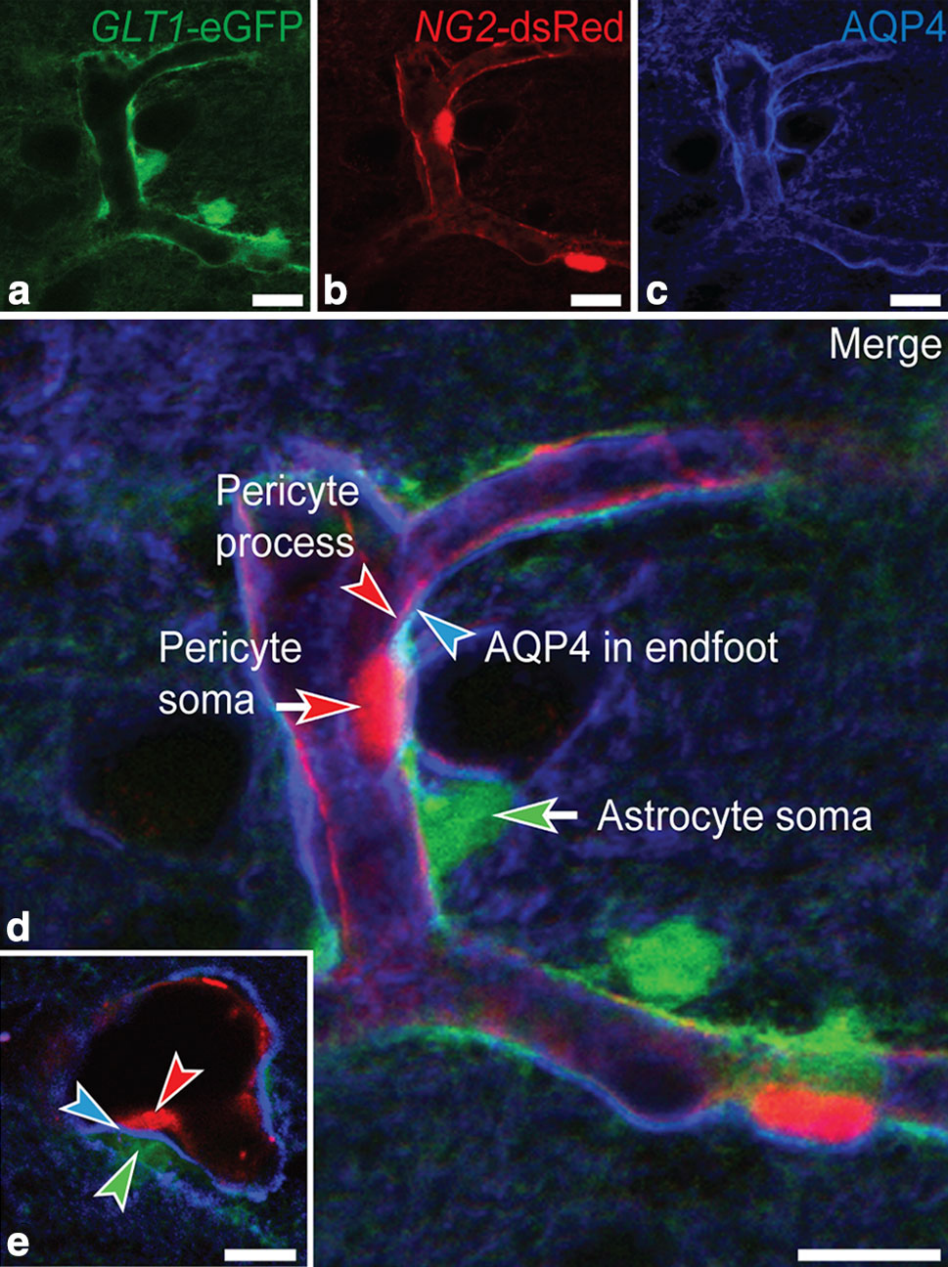
AQP4 immunofluorescence labeling is concentrated in astrocytic endfeet abutting pericytes. Micrographs from parietal cortex of *GLT1*-eGFP/*NG2*-DsRed transgenic double reporter mice showing the spatial relationship between astrocytes (**a**), pericytes (**b**), and AQP4 immunofluorescence labeling (**c**). **d** Astrocytic cell bodies (*green arrow*) and their perivascular endfeet (*green arrowhead*) ensheathe pericyte cell bodies (*red arrow*) and processes (*red arrowhead*). The AQP4 immunofluorescence signal appears particularly strong in astrocytic endfeet adjacent to pericyte cell bodies and processes. **e** Transverse section of a capillary branch showing distinct AQP4 immunofluorescence (*blue arrowhead*) between a pericyte (*red arrowhead*) and the eGFP-filled cytoplasm of an astrocytic endfoot process (*green arrowhead*), corresponding to the endfoot membrane.* Scale bars* 10 μm

To readily distinguish processes of astrocytes from those of pericytes at the light microscopical level, we generated a novel transgenic double reporter mouse. *GLT1*-eGFP BAC reporter mice expressing eGFP in astrocytes (Regan et al. 2007) were crossed with *NG2*-DsRed BAC reporter mice, which express DsRed in pericytes (Zhu et al. 2008), including those of brain capillaries (Iliff et al. 2012). AQP4 immunofluorescence labeling of the *GLT1*-eGFP/*NG2*-DsRed transgenic mice revealed strong AQP4 labeling in perivascular astrocytic processes adjacent to pericytes (Fig. 1a–e).

**Fig. 2:**
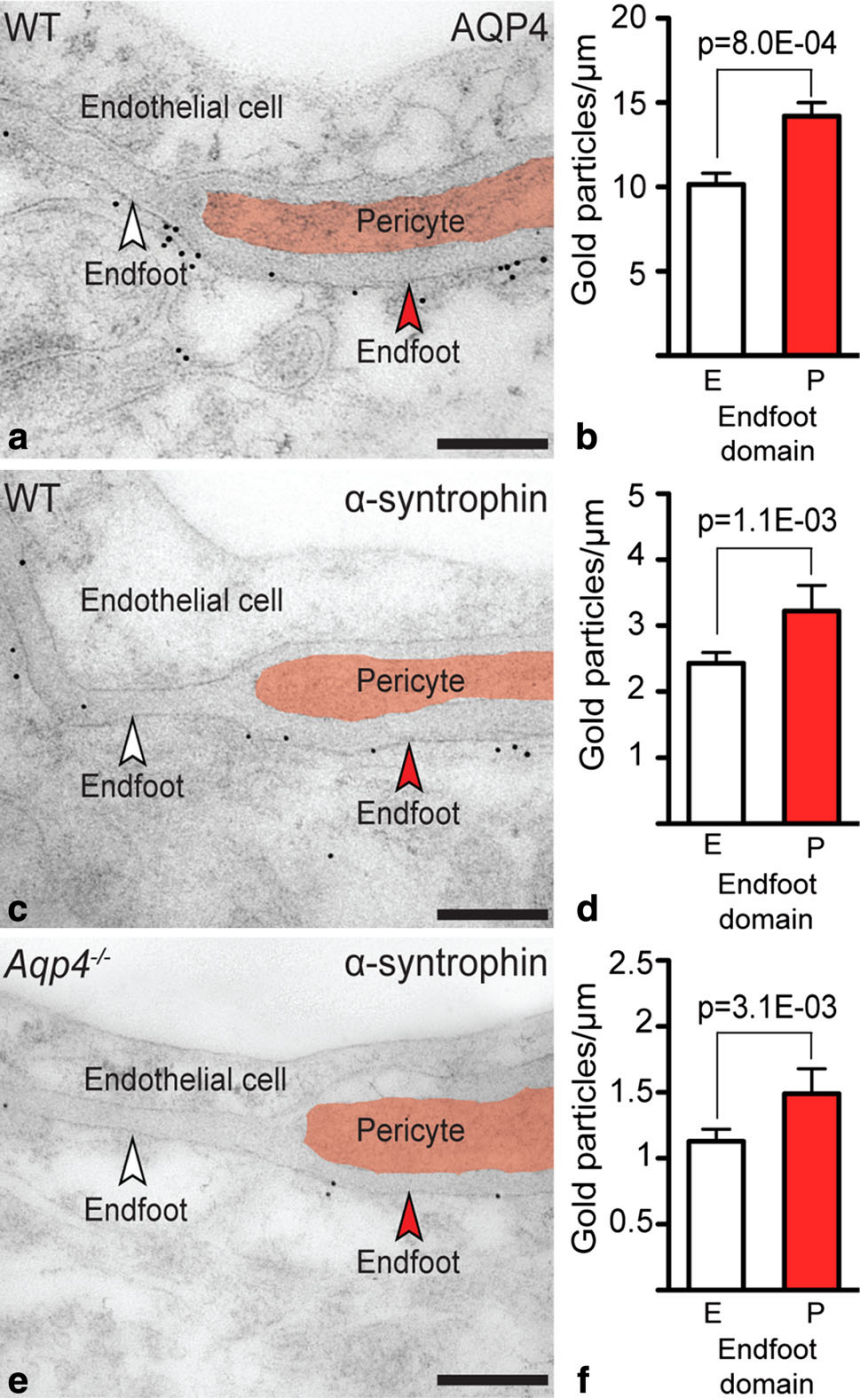
Immunogold labeling of AQP4 and α-syntrophin is concentrated in astrocytic endfoot membrane domains abutting pericytes. **a** Electron micrograph showing distribution of AQP4 immunogold labeling in parietal cortex of a wild-type (WT) mouse. Gold particles signaling AQP4 are clustered over astrocytic endfoot membranes, including membrane domains facing endothelial cells (E) and pericytes (P, marked *red*; *white* and *red arrowheads*, respectively). **b** Quantitative analysis of AQP4 immunogold labeling over astrocytic endfoot membrane domains. The density of gold particles over endfoot membranes abutting pericytes (P) is 40 % higher than over those facing endothelial cells (E). *P* value is indicated (Poisson mixed models, cf. Materials and methods; see “Results” for number of observations). **c** Electron micrograph showing immunogold staining of α-syntrophin of a WT mouse. Labels as in (**a**). **d** Quantitative analysis reveals 33 % higher density of α–syntrophin signaling gold particles over endfoot domains facing pericytes (P) compared to those facing endothelial cells (E). **e** α–Syntrophin immunogold labeling of an *Aqp4* ^−*/*−^ mouse. Labels as in (**a**). **f** The relative differences in α–syntrophin labeling density between endfoot membrane domains is preserved after deletion of *Aqp4*, being 32 % higher over membranes facing pericytes than over those facing endothelial cells.* Scale bars* 200 nm

Immunogold cytochemistry of C57BL/6 mice confirmed AQP4’s polarized distribution, with AQP4 signaling gold particles clustered over astrocytic endfoot membranes, in particular when in contact with pericytes (Fig. 2a). Quantitative analysis revealed that the density of gold particles was 40 % higher over membrane domains next to pericytes than over those facing endothelial cells (Fig. 2b; values were 14.20 ± 0.81, *n* = 38, and 10.15 ± 0.67, *n* = 72, respectively). The amount of unspecific labeling was revealed in corresponding membranes of *Aqp4* ^−/−^ mice and was only 1–2 % (values were 0.15 ± 0.04, *n* = 91, and 0.14 ± 0.11, *n* = 18).

We next investigated whether the enrichment of AQP4 in specific endfoot membrane domains was paralleled with increased expression of the AQP4-anchoring molecule α-syntrophin. Indeed, α-syntrophin immunogold reactivity was 33 % higher in membrane domains adjacent to pericytes versus endothelial cells (Fig. 2c, d; values were 3.22 ± 0.39, *n* = 61, and 2.43 ± 0.16, *n* = 215, respectively). Deletion of *Aqp4* reduced perivascular α-syntrophin labeling, as reported previously (Eilert-Olsen et al. 2012), but did not affect the pattern of α-syntrophin distribution in endfoot domains. Thus, in *Aqp4* ^−/−^ mice the α-syntrophin immunogold signal was still 32 % higher in endfoot membranes next to pericytes than over those next to endothelial cells (Fig. 2e, f; values were 1.49 ± 0.19, *n* = 53, and 1.13 ± 0.09, *n* = 198, respectively). The density of gold particles over endfoot membranes adjacent to pericytes and endothelial cells in mice lacking α-syntrophin was 0.25 ± 0.12, *n* = 30, and 0.17 ± 0.04, *n* = 146, respectively. Thus, unspecific labeling in the α-syntrophin experiments amounted to <8 % of the wild-type signal.

## Discussion

Polarized distribution of membrane proteins is essential for normal function of cells and tissues. A hallmark of glia is their polarization (Derouiche et al. 2012; Wolburg et al. 2011), with AQP4 being concentrated in glial endfoot membranes bordering liquor and paravascular compartments (Frigeri et al. 1995; Nielsen et al. 1997; Nagelhus et al. 1998). Loss of AQP4 from perivascular membranes occurs in epilepsy (Eid et al. 2005; Alvestad et al. 2013), stroke (Frydenlund et al. 2006), brain injury (Ren et al. 2013), and Alzheimer’s disease (Yang et al. 2011), but the mechanisms are poorly understood (Wolburg et al. 2011).

We now demonstrate that perivascular astrocytic endfoot membranes differ in their AQP4 expression. Specifically, the density of water channels is higher in membrane domains abutting pericytes than in those facing endothelial cells. Thus, the expression of AQP4 not only depends on which compartment the membrane faces, but also on the adjacent cell type.

The enrichment of AQP4 in membrane domains next to pericytes suggests that pericytes regulate AQP4 anchoring. Indeed, the AQP4-anchoring molecule α-syntrophin was also concentrated in membrane domains adjacent to pericytes. The clustering of α-syntrophin in these domains persisted after *Aqp4* deletion, indicating pericyte interaction with the DAPC independent of AQP4. The mechanisms by which pericytes control expression of endfoot scaffolding proteins are unknown. One possibility could be that pericytes secrete agrin, which binds α-dystroglycan (Wolburg et al. 2011). Altered agrin secretion from vascular cells may underlie loss of glial AQP4 polarization in disease.

The functional consequences of heterogeneous AQP4 expression along the vascular glial sheath are elusive. Brain–blood water diffusion distance is usually longer at sites where pericytes are interposed between the glial sheath and endothelial cells. It is conceivable that the higher aquaporin expression in glial membranes at such sites can partly compensate for the longer diffusion distance. Moreover, the enrichment of AQP4 in glial membranes adjacent to pericytes may serve to facilitate fluid transport between the paravascular space and the brain parenchyma. Notably, CSF recycles into the brain along paravascular spaces and enters the neuropil by AQP4-dependent mechanisms (Iliff et al. 2012). Finally, an intriguing possibility could be that the AQP4 supramolecular complex serves important signaling functions and thus is targeted to glial membranes adjacent to contractile vascular cells.

Platelet-derived growth factor B (PDGF-B) retention motif knockout mice lack pericytes and show reduced expression of perivascular AQP4 and α-syntrophin (Armulik et al. 2010). However, the mutant mice have vascular abnormalities that indirectly could affect AQP4 distribution, cf. “Introduction”. In the present study, we have provided evidence supporting the concept that pericytes regulate AQP4 anchoring and polarization in normal mice. Our discovery that astrocytic processes in contact with pericytes are equipped with specialized membrane domains adds complexity to the concept of astrocyte polarization and may pave the way for new understanding of mechanisms underlying loss of polarization in disease.
